# Predictors of Response to Treatment With Anti-calcitonin Gene-Related Peptide (CGRP) Antibodies in Real-World Patients With Episodic Migraine: A Two- and Four-Month Prospective Study

**DOI:** 10.7759/cureus.80345

**Published:** 2025-03-10

**Authors:** Abouch Krymchantowski, Carla Jevoux, Raimundo P Silva-Néto, Adriana A Soares, Maria Lucia Vellutini Pimentel, Ana Gabriela Krymchantowski, Hilton Mariano da Silva Júnior, Ervin Michelstaedter Cotrik

**Affiliations:** 1 Department of Post-graduation in Neurology, Headache Center of Rio, Rio de Janeiro, BRA; 2 Department of Neurology, Headache Center of Rio, Rio de Janeiro, BRA; 3 Department of Neurology, Hospital Municipal Miguel Couto, Rio de Janeiro, BRA; 4 Department of Neurology, Universidade Federal do Delta do Parnaíba, Delta do Parnaíba, BRA; 5 Department of Post-graduation in Neurology, Pontifícia Universidade Católica do Rio de Janeiro (PUC-RJ), Rio de Janeiro, BRA; 6 Department of Neurology, Pontifícia Universidade Católica de Campinas (PUC-Campinas), Campinas, BRA; 7 Department of Neurology, Universidade Estadual de Campinas, Campinas, BRA

**Keywords:** headache, migraine, monoclonal antibodies anti-cgrp, monthly headache days, treatment

## Abstract

Background: Monoclonal antibodies (mAbs) for anti-calcitonin gene-related peptide (CGRP) have emerged as an effective and well-tolerated option for alleviating migraine burden and improving patients’ quality of life. There is limited understanding of the specific clinical and biological predictors that forecast sustained response to anti-CGRP antibodies in real-world episodic migraine patients. This study was designed to estimate the proportion and potential predictors of response (≥50% response rate) at two months and four months in real-world patients with episodic migraine who received this therapy as their only preventive treatment.

Method: This is an open prospective study carried out in consecutive episodic migraine patients (International Classification of Headache Disorders (ICHD)-3) with six to 10 monthly headache days (MHD), seen for the first time from January 2023 to May 2024 in a tertiary center, to whom a monoclonal antibody anti-CGRP (fremanezumab or galcanezumab) was prescribed as the only preventive treatment. Sixty-three patients fulfilled the eligibility criteria. The patients were evaluated in long-lasting initial consultations, and follow-up visits were carried out after two and four months. Data was collected using a semi-structured proforma and a detailed headache diary.

Results: There was a significant reduction in the migraine frequency as measured by MHD from baseline to two months and four months after intervention (P=0.000, 8.85±1.17 days at baseline to 6.39±3.60 at two months and 6.35±3.25 at four months). The reduction in MHD was significantly higher among those who had a normal BMI as compared to the participants who were overweight (P=0.000) and those who had unilateral headaches (P=0.013) and severe osmophobia during attacks (P=0.035). Approximately 39.7% (n=25) of participants achieved a ≥50% reduction in MHD at both two and four months.

Conclusion: Normal BMI was found to be significantly associated with a reduction in migraine frequency of >50%, whereas normal BMI, unilateral headache, and severe osmophobia were significantly associated with a mean MHD reduction. Further controlled studies with several other factors predicting response to anti-CGRP mAbs are warranted.

## Introduction

Migraine is a highly prevalent neurological disease characterized by incapacitating headache attacks with associated features. Oral preventive migraine treatments are often non-specific, non-selective, poorly tolerated, and associated with high dropout rates [1]. By combining a good tolerability profile with promising efficacy, monoclonal antibodies (mAbs) that target the calcitonin gene-related peptide (CGRP), the first specific migraine prophylactic medications, are transforming migraine prophylaxis [2].

Anti-CGRP monoclonal antibodies are unique due to their superior tolerability and efficacy compared to standard therapies, with a significant proportion of patients achieving a ≥50% reduction in monthly migraine days [3]. Studies employing the CGRP mAbs galcanezumab and fremanezumab showed 50% response rates of up to 62% in episodic migraine (EM) [4-6] and 41% in chronic migraine (CM) [7,8]. Even in patients who had previously experienced several unsuccessful attempts at preventative treatment, CGRP mAbs generated a >50% response in one-third of cases [9-11].

Anti-CGRP mAbs have shown efficacy in reducing migraine burden and improving quality of life [12]. Although the pivotal registration trials failed to identify unambiguous predictors of response, knowing that is a desire of clinicians involved in migraine treatment. A higher response to treatment was positively correlated with unilateral pain localization and triptan responsiveness in a few real-world investigations [13-17]. Conversely, a poorer response was linked to psychiatric comorbidities, a lengthy illness duration, and a significant number of prior unsuccessful preventative interventions [13,14].

Many clinical and demographic factors, including age, sex, body mass index, basal migraine frequency and disability, pain intensity, and side effects, allodynia, dopaminergic symptoms, triptan reaction, psychiatric comorbidities, and personality traits, have been linked to responsiveness to anti-CGRP mAbs, according to some research. Variations in the populations examined, sample sizes, research methodologies, and clinical outcomes examined may all contribute to the findings' variability [13,14,16,18-20].

Despite clinical trial data, real-world predictors of response to anti-CGRP mAbs remain unclear, necessitating further investigation. To date, it has not been possible to define people who respond well to mAb prophylaxis and those who do not, which could aid in more individualized treatment strategies for patients with episodic migraine. Furthermore, even though mAbs are successful in pivotal and clinical studies, it is uncertain if real-world participants from tertiary centers, who typically get polypharmacy, would respond well to the use of mAbs alone.

We designed this study to examine the proportion and potential predictors of response (≥50% response rate) at two and four months specifically in patients with episodic migraine from a South American country where the treatment reality may provide insights into the sociodemographic or clinical profiling of responders to anti-CGRP mAbs.

## Materials and methods

Study design

The study was an open-label prospective study carried out in a single tertiary center, with consecutive episodic migraine patients seen for the first time from January 2023 to May 2024. The Ethics in Research Involving Human Subjects Committee at the Federal University of Piauí and the National Ethics in Research System issued approval for the conduct of the study, and the approval number is 5076361.

Inclusion criteria

The study included all patients with the diagnosis of episodic migraine according to the International Classification of Headache Disorders (ICHD)-3, with a baseline frequency of six to 10 headache days/month during the previous three months, who received a monoclonal antibody anti-CGRP (fremanezumab or galcanezumab, the only two mAbs available in Brazil) as the only preventive treatment. All patients were prescribed rizatriptan or eletriptan plus a non-steroidal anti-inflammatory drug (NSAID) for the acute treatment of attacks in a maximum intake of two days per week.

Exclusion criteria

Patients using preventive medications at the time of inclusion, those who had any preventive agents during the previous three months, and those who have used onabotulinumtoxin A in the last six months were excluded to avoid the confounding effect of these interventions. In addition, women not using contraceptive methods or planning to get pregnant within the following six months were excluded to prevent the mAbs from affecting conception.

Sample size

From a previous study, the proportion of response (≥50% response rate) at two months in patients with episodic migraine was 20.5% [5]. Assuming an 11% absolute error and a 95% confidence level, the required minimum sample size was calculated as 52.

Data collection

Sixty-three subjects met the eligibility requirements of the 273 patients who had chronic or episodic migraine and were evaluated for the first time within the same period. Following the signing of the informed consent form, each patient underwent a comprehensive history of headaches and a thorough physical and neurological examination. The patients were seen by trained neurologists, who gathered data on sociodemographics, headache and migraine characteristics, past and present migraine treatments, comorbidities, and concurrent medications. They also received a detailed headache diary filled with the headache frequency, severity, and onset features; preceding symptoms; triggers; medication taken; and time of relief, as well as pain-free status. Patients were seen after two and four months. Responders had a reduction in monthly headache days of 50% or higher. Non-responders had a reduction in monthly headache days of less than 50%.

Statistical analysis

Data were checked for completeness and consistency. Data was analyzed using SPSS software version 21 (IBM Corp., Armonk, NY, USA). Descriptive statistics like percentages and means were used. Inferential analysis was done using the chi-square test, unpaired t-test, and repeated measures ANOVA. A P-value of less than 0.05 was taken as statistically significant.

## Results

Table 1 shows the baseline characteristics of the study participants. The majority, 33 (52.4%) of the participants, received galcanezumab 240 mg as a loading dose and 120 mg/month, and to the rest of the subjects, fremanezumab 225 mg/month was prescribed for preventive treatment of migraine. Most participants (81.2%, n=51) were female. Thirty-two (50.8%) of the participants were in the age group of 41 to 50 years, and the mean age of the participants was 45.46±9.26 years. Approximately 45 participants (71.4%) exhibited a normal BMI, with a mean BMI of 24.17±1.22. Medicated comorbidity was present in two-thirds, 42 (66.7%) of the participants. Thirty (47.6%) of the participants reported frequent insomnia. Visual aura was reported by 10 (15.9%) of the participants. In this study, 26 (41.3%) participants had unilateral headaches. Severe nausea, 27 (42.9%); vomiting, seven (11.1%); severe photophobia, 45 (71.4%); severe phonophobia, nine (14.3%); and severe osmophobia, 27 (42.9%) during attacks were the associated features reported by the patients. Unilateral headache was seen in 26 (41.3%), and rapid progression of headache was present in 29 (46.0%) participants. Topiramate, tricyclic antidepressants, and beta blockers were used by 33 (52.4%), 28 (44.4%), and 14 (22.2%) of the participants, respectively. The majority of the patients, 26 (41.3%), had a history of migraine for 16 to 30 years, and 25 (39.7%) of the participants had had migraine for longer than 30 years. The mean duration of migraine was 27.29±10.11 years.

**Table 1:** Baseline characteristics of the study participants TCA: tricyclic antidepressant

Variable	Category	Frequency	Percent
Monoclonal antibody used	Fremanezumab 225/mo	30	47.6
	Galcanezumab 240-120/mo	33	52.4
Gender	Male	12	19.0
	Female	51	81.0
Age group	18 to 40 years	12	19.0
	41 to 50 years	32	50.8
	More than 50 years	19	30.2
BMI	Normal (less than 25.00)	45	71.4
	Overweight (25 to 29.99)	18	28.6
Exercise	2d/week	7	11.1
	3d/week	14	22.2
	4d/week	6	9.5
	5d/week	1	1.6
	6d/week	1	1.6
	No	34	54.0
Medicated comorbidity	No	21	33.3
	Yes	42	66.7
Quality of sleep	Good	28	44.4
	Interrupted sleep	5	7.9
	Insomnia	30	47.6
Migraine with aura	No	53	84.1
	Yes	10	15.9
Unilateral headache	No	37	58.7
	Yes	26	41.3
Severe nausea	No	36	57.1
	Yes	27	42.9
Vomiting	No	56	88.9
	Yes	7	11.1
Photophobia	No	18	28.6
	Yes	45	71.4
Phonophobia	No	54	85.7
	Yes	9	14.3
Osmophobia	No	36	57.1
	Yes	27	42.9
Rapidly progression	No	34	54.0
	Yes	29	46.0
Previous use of Botox	No	34	54.0
	Yes	29	46.0
Previous use of topiramate	No	30	47.6
	Yes	33	52.4
Previous use of TCA	No	35	55.6
	Yes	28	44.4
Previous use of Beta Blockers	No	49	77.8
	Yes	14	22.2
Time with migraine	Less than and equal to 15 years	12	19.0
	16 years to 30 years	26	41.3
	More than 30 years	25	39.7
Age	Mean (SD)	45.46	9.26
BMI	Mean (SD)	24.17	1.22
Time with migraine in years	Mean (SD)	27.29	10.11

Medicated comorbidity (P=0.001) was significantly higher among those who received fremanezumab 225 mg/month for the treatment of migraine, but it was coincidental. The prevalence of severe nausea (P=0.013), vomiting (P=0.007), and phonophobia (P=0.018) were significantly higher among those who were given galcanezumab 240-120 mg/month. The distribution of other characteristics was not significantly different between the two groups of intervention (Table 2).

**Table 2:** Distribution of baseline characteristics between two groups of intervention (chi-square test and unpaired t test) TCA: tricyclic antidepressant

Variable	Category	Fremanezumab 225/mo	Galcanezumab 240-120/mo	p-value
Gender	Male	5 (16.7)	7 (21.2)	0.646
	Female	25 (83.3)	26 (78.8)	
Age group	18 to 40 years	5 (16.7)	7 (21.2)	0.092
	41 to 50 years	12 (40.0)	20 (60.6)	
	More than 50 years	13 (43.3)	6 (18.2)	
BMI	Normal (less than 25.00)	20 (66.7)	25 (75.8)	0.425
	Overweight (25 to 30.0)	10 (33.3)	8 (24.2)	
Exercise	2d/week	4 (13.3)	3 (9.1)	0.050
	3d/week	2 (6.7)	12 (36.4)	
	4d/week	3 (10.0)	3 (9.1)	
	5d/week	0 (0.0)	1 (3.0)	
	6d/week	0 (0.0)	1 (3.0)	
	No	21 (70.0)	13 (39.4)	
Medicated comorbidity	No	4 (13.3)	17 (51.5)	0.001
	Yes	26 (86.7)	16 (48.5)	
Quality of sleep	Good	10 (33.3)	18 (54.6)	0.203
	Interrupted sleep	4 (13.3)	1 (3.0)	
	Insomnia	16 (53.4)	14 (42.4)	
Migraine with aura	No	24 (80.0)	29 (87.9)	0.393
	Yes	6 (20.0)	4 (12.1)	
Unilateral headache	No	18 (60.0)	19 (57.6)	0.845
	Yes	12 (40.0)	14 (42.4)	
Severe nausea	No	22 (73.3)	14 (42.4)	0.013
	Yes	8 (26.4)	19 (57.6)	
Vomiting	No	30 (100.0)	26 (78.8)	0.007
	Yes	0 (0.0)	7 (21.2)	
Photophobia	No	12 (40.0)	6 (18.2)	0.056
	Yes	18 (60.0)	27 (81.8)	
Phonophobia	No	29 (96.7)	25 (75.8)	0.018
	Yes	1 (3.3)	8 (24.2)	
Osmophobia	No	19 (63.3)	17 (51.5)	0.344
	Yes	11 (36.7)	16 (48.5)	
Rapidly progression	No	18 (60.0)	16 (48.5)	0.360
	Yes	12 (40.0)	17 (51.5)	
Previous use of Botox	No	21 (70.0)	25 (75.8)	0.607
	Yes	9 (30.0)	8 (24.2)	
Previous use of topiramate	No	17 (56.7)	18 (54.5)	0.866
	Yes	13 (43.3)	15 (45.5)	
Previous use of TCA	No	15 (50.0)	15 (45.5)	0.718
	Yes	15 (50.0)	18 (54.5)	
Previous use of Beta Blockers	No	26 (86.7)	23 (69.7)	0.106
	Yes	4 (13.3)	10 (30.3)	
Time with migraine in years	Less than and equal to 15 years	5 (16.7)	7 (21.2)	0.891
	16 years to 30 years	13 (43.3)	13 (39.4)	
	More than 30 years	12 (40.0)	13 (39.4)	
Age	Mean (SD)	47.33 (9.38)	43.76 (8.96)	0.127
BMI	Mean (SD)	24.38 (1.23)	23.99 (1.20)	0.209
Time with migraine in years	Mean (SD)	27.53 (9.69)	27.06 (10.62)	0.854

**Figure 1:**
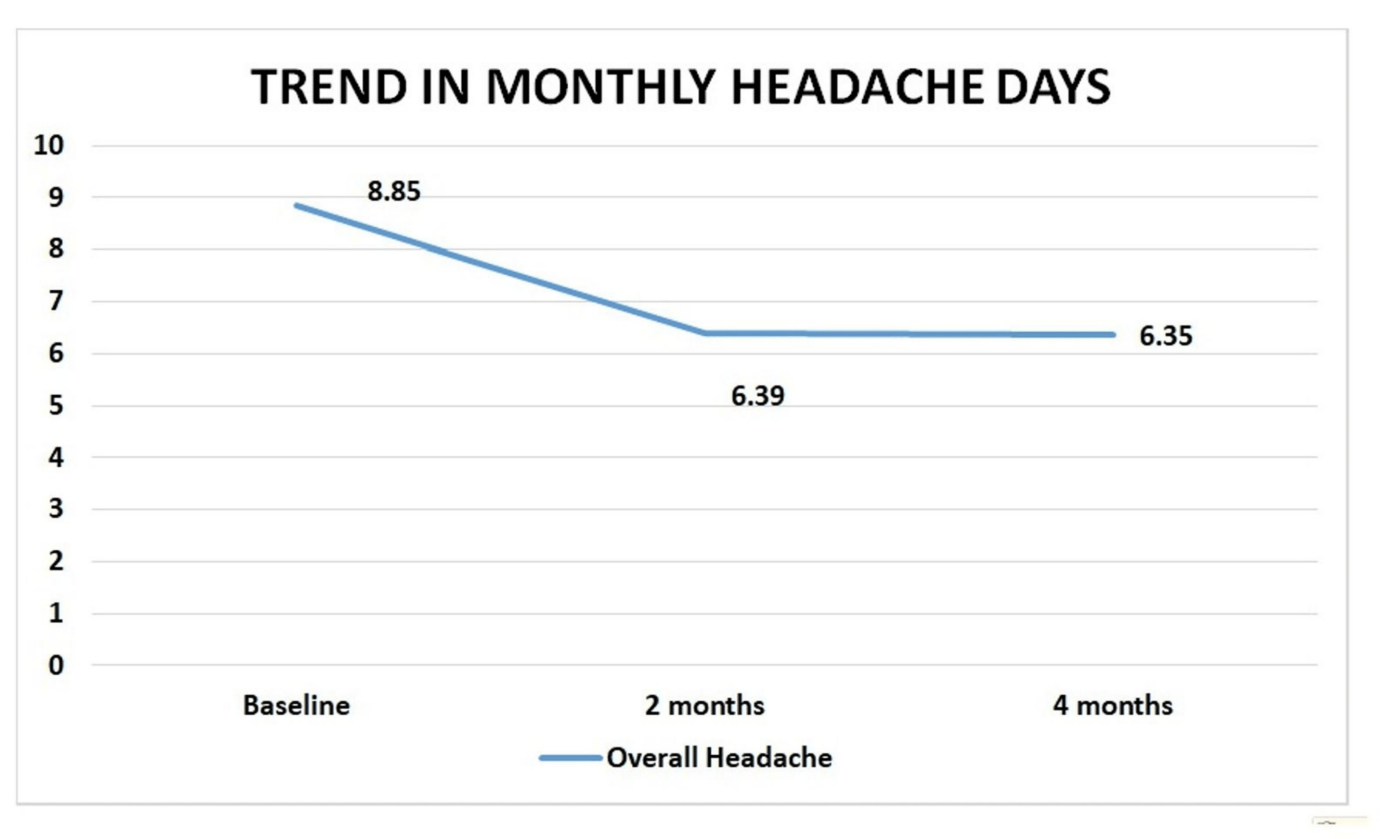
Change in migraine frequency over time

Figure 1 and Table 3 showed that there was a significant reduction in migraine frequency as measured by the MHD from baseline to two months and four months after intervention (P=0.000, 8.85±1.17 days at baseline to 6.39±3.60 at two months and 6.35±3.25 at four months). There was no significant association found between reduction in MHD and any other characteristics.

**Table 3:** Change in migraine frequency (repeated measures ANOVA) *P-value is less than 0.05 is considered as statistically significant mAb: monoclonal antibody; Galca: galcanezumab; Frema: fremanezumab

Variable	Category	Baseline (Mean ± SD)	At two months after intervention (Mean ± SD)	At four months after intervention (Mean ± SD)	F value	P value
Total		8.85±1.17	6.39±3.60	6.35±3.25	21.643	0.000^*^
mAb and dose	Frema 225/mo	8.69±1.16	6.92±3.39	7.08±3.21	1.396	0.243
	Galca 240-120/mo	9±1.19	5.89±3.77	5.68±3.19		
Gender	Male	9±1.41	5.44±3.81	5±2.60	1.142	0.290
	FEMALE	8.82±1.13	6.58±3.56	6.62±3.32		
AGE	18 TO 40 YEARS	9.4±1.08	6.9±3.96	6.9±3.60	0.572	0.568
	41 TO 50 YEARS	8.89±1.09	6.07±3.89	5.74±3.14		
	MORE THAN 50 YEARS	8.47±1.28	6.59±3.00	7±3.20		
BMI	NORMAL	8.92±1.23	5.22±3.50	5.35±3.23	15.08	0.000*
	OVERWEIGHT	8.71±1.05	8.94±2.28	8.53±2.04		
UNILATERAL HEADACHE	NO	8.77±1.12	7.39±3.44	7.32±3.35	6.576	0.013*
	YES	8.96±1.26	5.04±3.42	5.04±2.64		
SEVERE NAUSEA	NO	8.93±1.14	7.03±3.90	7.1±3.43	3.511	0.067
	YES	8.75±1.23	5.58±3.06	5.42±2.80		
VOMITING	NO	8.91±1.18	6.28±3.59	6.32±3.26	0.055	0.816
	YES	8.43±1.13	7.14±3.85	6.57±3.41		
PHOTOPHOBIA	NO	8.06±1.06	7.25±3.70	7.31±3.88	0.549	0.462
	YES	9.18±.1.06	6.03±3.54	5.95±2.90		
PHONOPHOBIA	NO	8.91±1.13	6.67±3.68	6.58±3.11	1.992	0.164
	YES	8.56±1.42	5±2.92	5.22±3.87		
OSMOPHOBIA	NO	8.66±1.23	7.45±3.82	7.24±3.38	4.703	0.035*
	YES	9.08±1.08	5.16±2.93	5.32±2.81		
RAPID PROGRESSION	NO	8.64±1.22	7.07±3.32	7.32±3.45	2.865	0.096
	YES	9.08±1.09	5.65±3.79	5.31±2.70		

**Figure 2:**
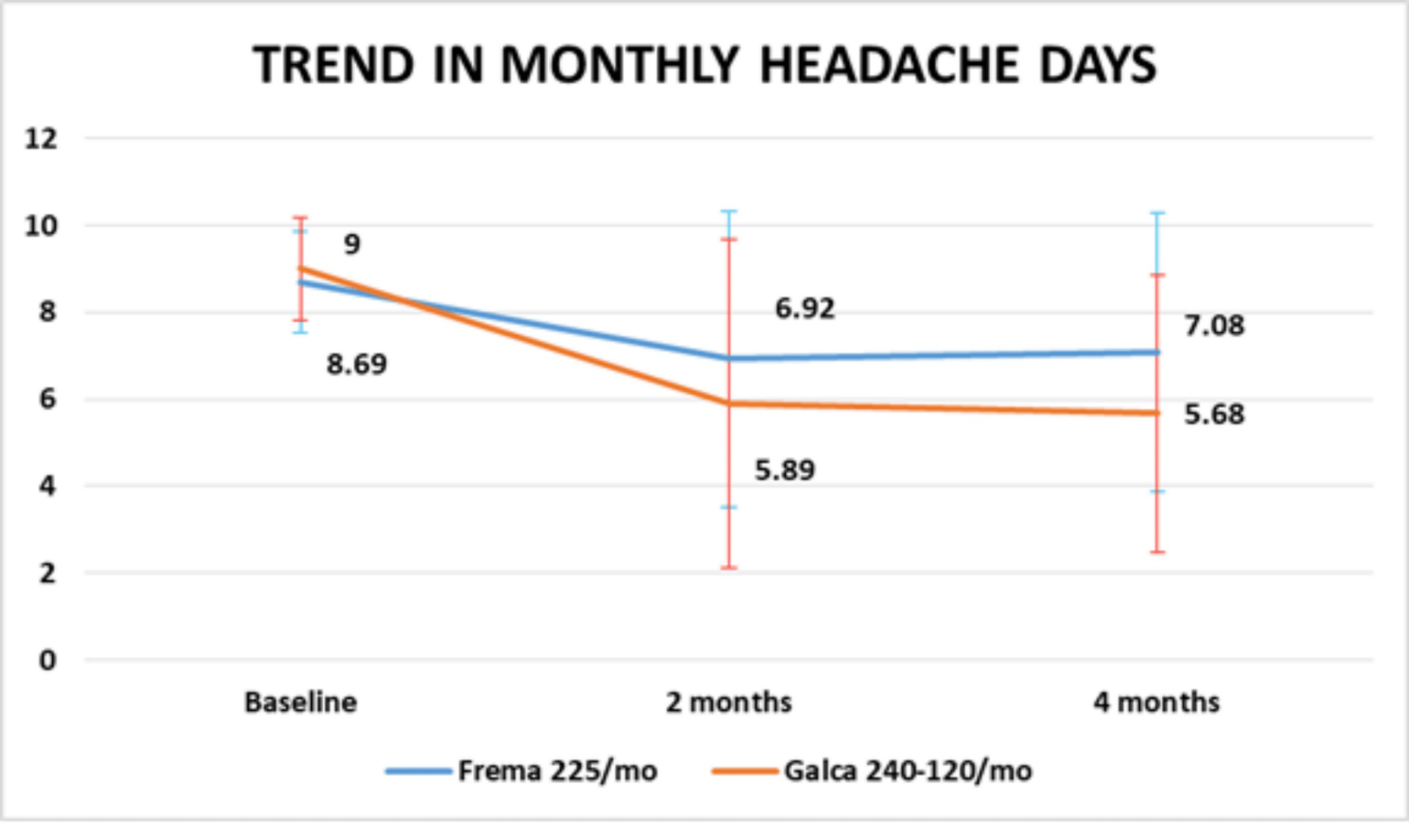
Change in migraine frequency with mAb mAb: monoclonal antibody; Galca: galcanezumab; Frema: fremanezumab

There was no significant difference (P=0.243) in the reduction of MHD between the two different types of mAbs given for treatment of migraine (Figure 2).

**Figure 3:**
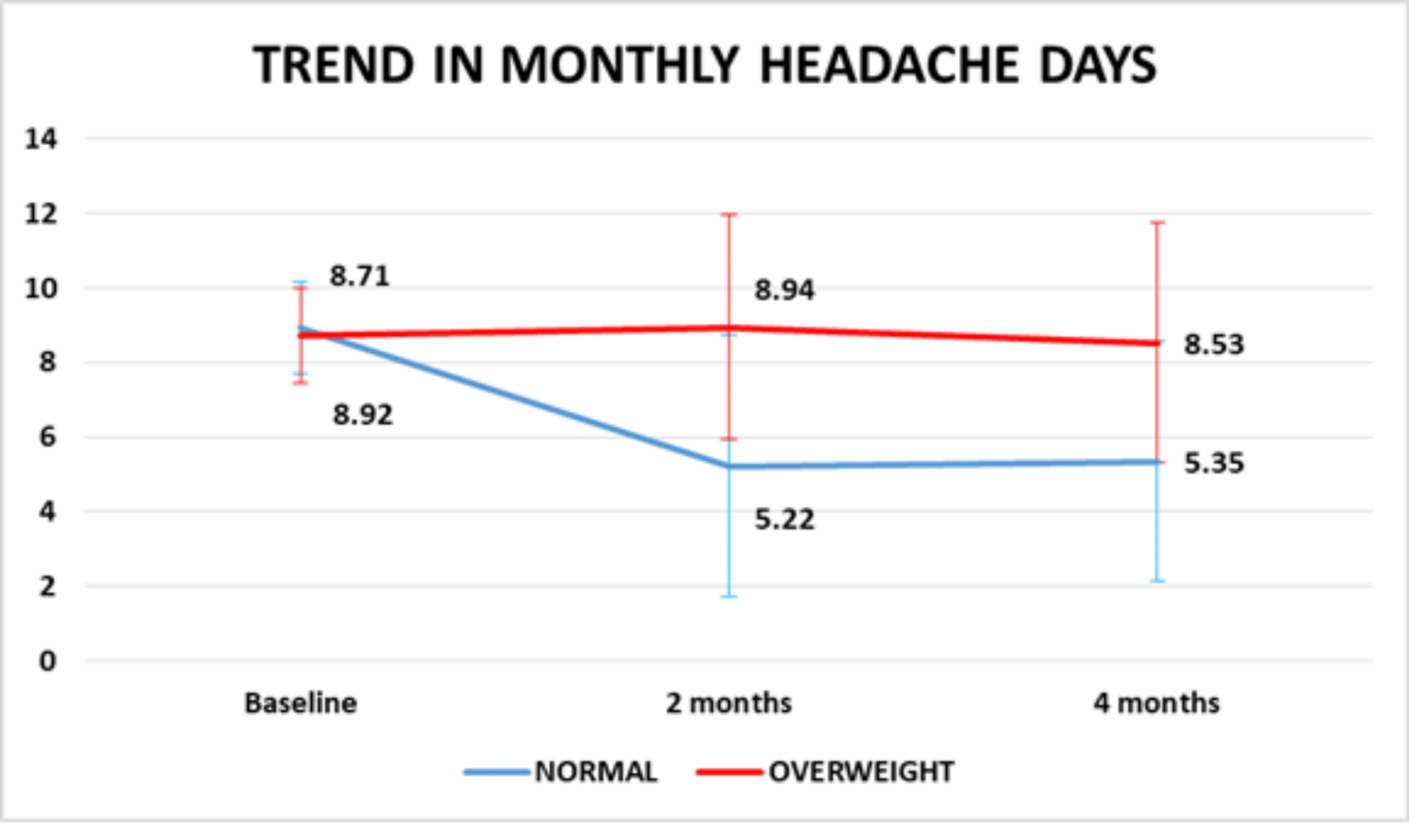
Change in migraine frequency with BMI (P value is less than 0.05)

The reduction in the MHD was significantly higher among those who had normal BMI as compared to the participants who were overweight (P=0.000) (Figure 3).

**Figure 4:**
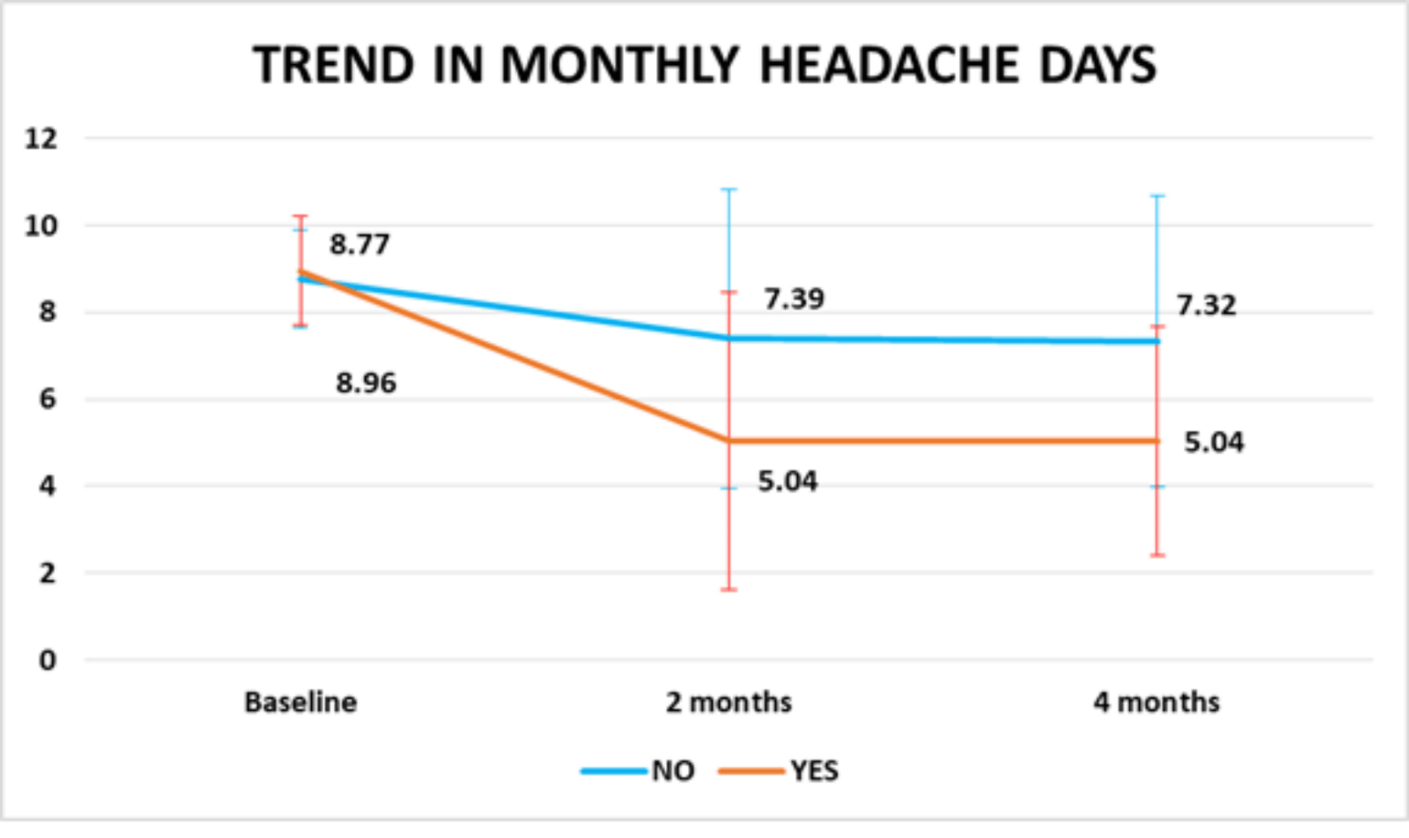
Change in migraine frequency with unilateral headache (P value is less than 0.05)

**Figure 5:**
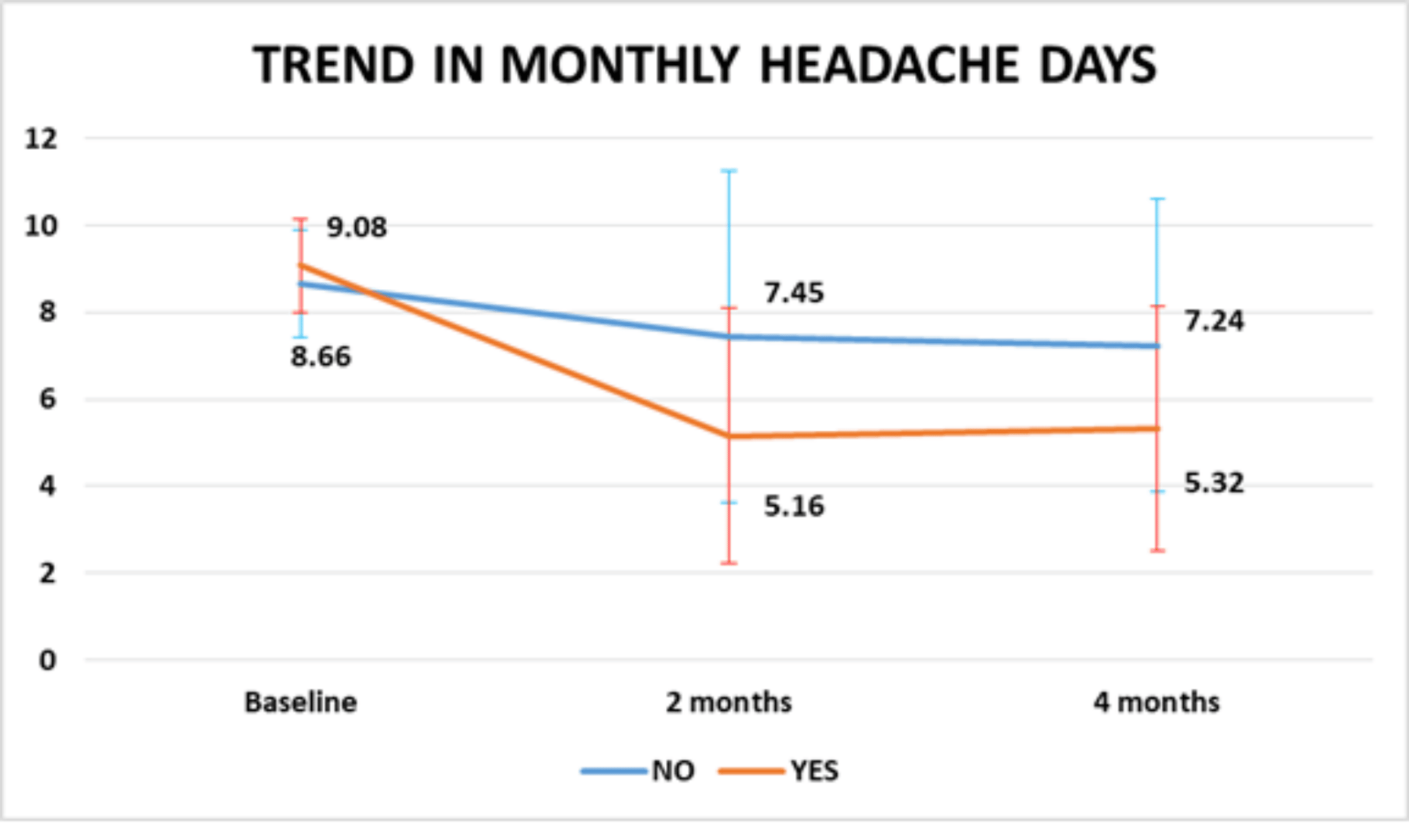
Change in migraine frequency with osmophobia (P value is less than 0.05)

The presence of unilateral headache (P=0.013) and severe osmophobia during attacks (P=0.035) was significantly associated with a higher reduction in MHD (Figure 4 and Figure 5).

**Figure 6:**
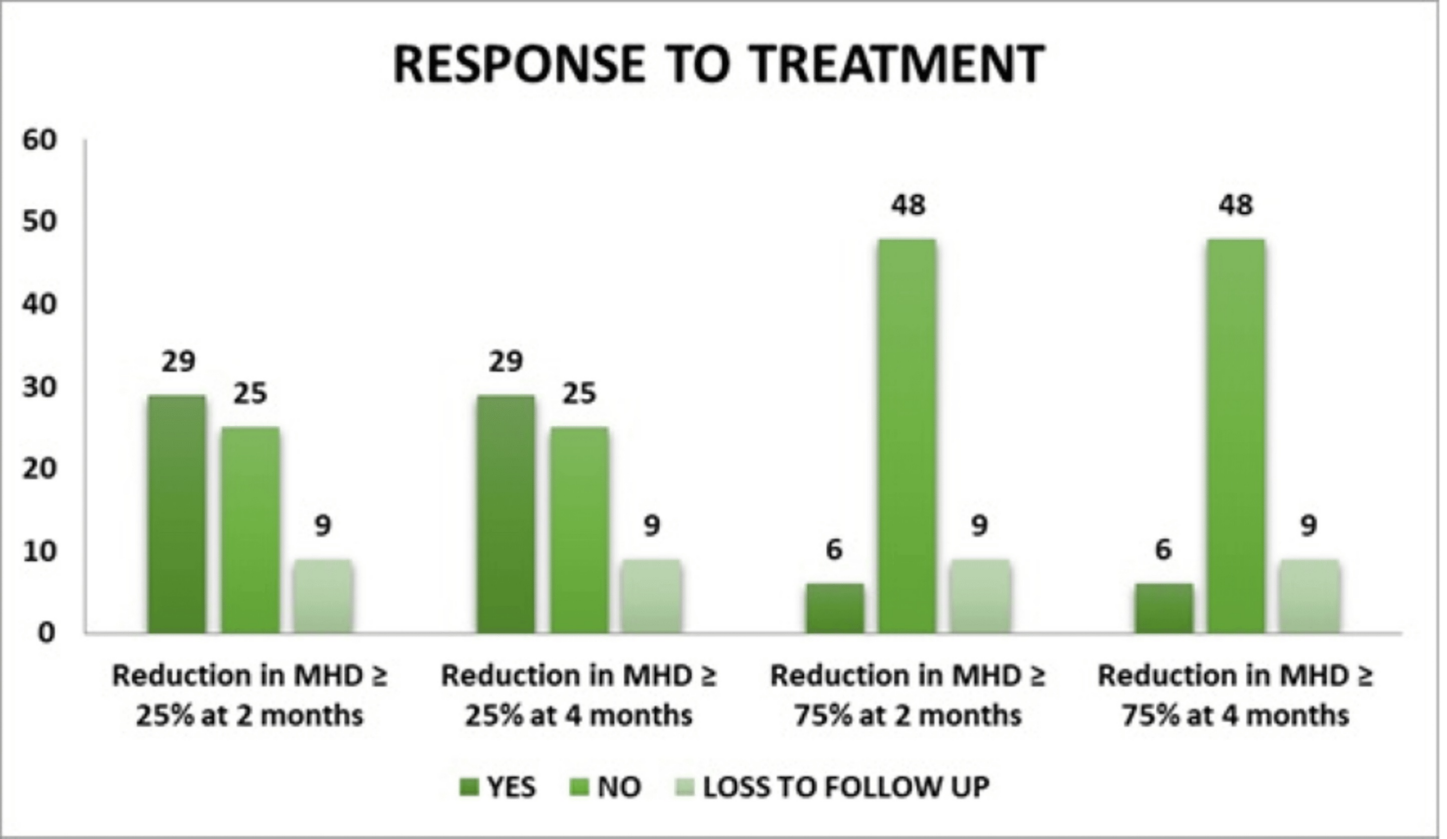
Response to treatment MHD: monthly headache days

At two months, 25 (39.7%) of the participants experienced a ≥50% decrease in monthly headache days. Twenty-five (39.7%) of the participants experienced a 50% reduction in monthly headache days after four months. Twenty-nine participants (46.0%) experienced a reduction of at least 25% in monthly headache days at both two months and four months. Around six participants (9.5%) experienced a reduction of 75% or more in monthly headache days at both two and four months (Figure 6).

Only normal BMI (OR=29.54(95%CI=3.51-248.58)) and presence of unilateral headache (OR=3.94(95%CI=1.26-12.33)) were found to be significantly associated with ≥50% reduction in MHD at two months and four months (P<0.001) (Table 4 and Table 5).

**Table 4:** Reduction of monthly headache days ≥50% at two months (chi-square test) *P-value is less than 0.05 is statistically significant mAb: monoclonal antibody; Galca: galcanezumab; Frema: fremanezumab

Variable	Category	Yes n (%)	No n (%)	Loss to follow up n (%)	Odds ratio (95% CI)	P value
Total		25 (39.7)	29 (46.0)	9 (14.3)		
Age	18 to 40 Years	4 (33.3)	6 (50.0)	2 (16.7)	1.00	
	41 to 50 Years	15 (46.9)	12 (37.5)	5 (15.6)	1.88 (0.43-8.20)	0.404
	More than 50 years	6 (31.6)	11 (57.9)	2 (10.5)	0.82 (0.16-4.09)	0.809
BMI	Normal	24 (53.3)	13 (28.9)	8 (17.8)	29.54 (3.51-248.58)	0.001^*^
	Overweight	1 (5.6)	16 (88.9)	1 (5.6)	1.00	
Gender	Male	6 (50.0)	3 (25.0)	3 (25.0)	1.00	
	Female	19 (37.3)	26 (51.0)	6 (11.8)	0.37 (0.08-1.65)	0.190
mAb	Frema 225/Mo	9 (30.00)	17 (56.7)	4 (13.3)	1.00	
	Galca 240-120/Mo	16 (48.5)	12 (36.4)	5 (15.2)	2.52 (0.84-7.58)	0.100
Unilateral	No	10 (27.0)	21 (56.8)	6 (16.2)	1.00	
	Yes	15 (57.7)	8 (30.8)	3 (11.5)	3.94 (1.26-12.33)	0.02^*^
Severe nausea	No	12 (33.3)	18 (50.0)	6 916.7)	1.00	
	Yes	13 (48.1)	11 (40.7)	3 (11.1)	1.77 (0.60-5.25)	0.301
Vomiting	No	22 (39.3)	25 (44.6)	9 (16.1)	1.00	
	Yes	3 (42.9)	4 (57.1)	0 (0.0)	0.85 (0.17-0.42)	0.845
Photophobia	No	6 (33.3)	10 (55.6)	2 (11.1)	1.00	
	Yes	19 (42.2)	19 (42.2)	7 (15.6)	1.67 (0.50-5.51)	0.402
Phonophobia	No	19 (35.2)	26 (48.1)	9 (16.7)	1.00	
	Yes	6 (66.7)	3 (33.3)	0 (0.0)	2.74 (0.61-12.35)	0.190
Osmophobia	No	10 (27.8)	19 (52.8)	7 (19.4)	1.00	
	Yes	15 (55.6)	10 (37.0)	2 (7.4)	2.85 (0.94-8.63)	0.063
Rapid progression	No	10 (29.4)	18 (52.9)	6 (17.6)	1.00	
	Yes	15 (51.7)	11 (37.9)	3 (10.3)	2.45 (0.82-7.35)	0.108

**Table 5:** Reduction of monthly headache days ≥50% at four months (chi-square test) *P-value is less than 0.05 is statistically significant mAb: monoclonal antibody; Galca: galcanezumab; Frema: fremanezumab

Variable	Category	Yes n (%)	No n (%)	Loss to follow up n (%)	Odds ratio (95% CI)	P value
Total		25 (39.7)	29 (46.0)	9 (14.3)		
Age	18 to 40 Years	4 (33.3)	6 (50.0)	2 (16.7)	1.00	
	41 to 50 Years	15 (46.9)	12 (37.5)	5 (15.6)	1.88(0.43-8.20)	0.404
	More than 50 years	6 (31.6)	11 (57.9)	2 (10.5)	0.82(0.16-4.09)	0.809
BMI	Normal	24 (53.3)	13 (28.9)	8 (17.8)	29.54(3.51-248.58)	0.001^*^
	Overweight	1 (5.6)	16 (88.9)	1 (5.6)	1.00	
Gender	Male	6 (50.0)	3 (25.0)	3 (25.0)	1.00	
	Female	19 (37.3)	26 (51.0)	6 (11.8)	0.37(0.08-1.65)	0.190
mAb	Frema 225/Mo	9 (30.00)	17 (56.7)	4 (13.3)	1.00	
	Galca 240-120/Mo	16 (48.5)	12 (36.4)	5 (15.2)	2.52(0.84-7.58)	0.100
Unilateral	No	10 (27.0)	21 (56.8)	6 (16.2)	1.00	
	Yes	15 (57.7)	8 (30.8)	3 (11.5)	3.94(1.26-12.33)	0.02^*^
Severe nausea	No	12 (33.3)	18 (50.0)	6 916.7)	1.00	
	Yes	13 (48.1)	11 (40.7)	3 (11.1)	1.77(0.60-5.25)	0.301
Vomiting	No	22 (39.3)	25 (44.6)	9 (16.1)	1.00	
	Yes	3 (42.9)	4 (57.1)	0 (0.0)	0.85(0.17-0.42)	0.845
Photophobia	No	6 (33.3)	10 (55.6)	2 (11.1)	1.00	
	Yes	19 (42.2)	19 (42.2)	7 (15.6)	1.67(0.50-5.51)	0.402
Phonophobia	No	19 (35.2)	26 (48.1)	9 (16.7)	1.00	
	Yes	6 (66.7)	3 (33.3)	0 (0.0)	2.74(0.61-12.35)	0.190
Osmophobia	No	10 (27.8)	19 (52.8)	7 (19.4)	1.00	
	Yes	15 (55.6)	10 (37.0)	2 (7.4)	2.85(0.94-8.63)	0.063
Rapid progression	No	10 (29.4)	18 (52.9)	6 (17.6)	1.00	
	Yes	15 (51.7)	11 (37.9)	3 (10.3)	2.45(0.82-7.35)	0.108

## Discussion

Nearly two-fifths, 25 (39.7%) of the participants had a reduction of monthly headache days ≥50% at two months and four months, and the reduction of monthly headache days was significant from baseline to two months and four months after intervention.

Research on individuals with episodic migraines showed a similar result [4-6]. Galcanezumab, 120 mg, dramatically decreased mean migraine headache days in the study by Skljarevski et al. [5]. In the Stauffer et al. research, galcanezumab treatment significantly decreased monthly migraine headache days with 120 mg and 240 mg as compared to placebo [5]. In different research, the mean number of migraine days per month dropped in two dosages of fremanezumab from baseline to 12 weeks [6].

In studies conducted on patients with chronic migraine, galcanezumab and fremanezumab also dramatically decreased the number of monthly migraine days [7,8]. 20.5% of patients maintained at least 50% response after receiving 120 mg of galcanezumab for six consecutive months, compared to 19.2% who received 240 mg [5]. The 50% response rate for patients who got monthly doses of fremanezumab was 47.7%, while the 50% response rate for patients who received a single, higher dose was 44.4% [6]. The 50% reduction in MHDs in CM patients was around 41% [7,8]. Notably, there are significant variations in the endpoint determination since different studies have different trial designs and statistical analyses [4-8]. Even in patients who had previously had several unsuccessful attempts at prophylactic medication, CGRP(-R) mAbs generated a >50% response in one-third of cases [9-11].

During the chosen observation period, 16% of the EM patients experienced no migraines, and 39% experienced a ≥75% reduction in monthly migraine days (MMD) in the study by Broessner G et al. [21]. In the study by Cullum CK et al, real-world data using CGRP(-R) mAbs in clinical practice show comparable outcomes. Following three months of treatment plan, CM patients experienced a 50% decrease in MMD [22]. While up to one-third of patients do not react to mAbs treatment, 20% of patients get a very substantial benefit with a reduction of >75% in MMD and a notable improvement in quality of life [22].

When considering the baseline characteristics, only normal BMI was significantly associated with a ≥50% reduction in migraine frequency, with 53% of normal BMI participants achieving this reduction compared to 5.6% of overweight individuals. The reduction in the migraine MHD was significantly higher among those who had a normal BMI as compared to the participants who were overweight (p=0.000). Likewise, a recent systematic review study done by Oleveira et al. found that obesity is a strong negative predictor for anti-CGRP mAbs therapy in chronic migraine patients [23]. Meanwhile, studies indicate that BMI is linked to various CNS pathologies, including an increased risk of spontaneous intracerebral hemorrhage [24]. Given the complex interplay of obesity, vascular pathology, and undiagnosed systemic conditions, further studies are warranted to establish a clearer pathophysiological link and enhance predictive measures for at-risk populations. Greater emphasis should be placed on weight management as a preventive strategy. Integrating this perspective can enhance the understanding of BMI’s role in CNS pathologies, providing a stronger foundation for preventive strategies and future research directions.

According to Barbanti et al., obesity was found to be a poor predictor of CM patients' responsiveness to anti-CGRP mAbs. One explanation is that current anti-CGRP mAb treatments may not be able to adequately counteract the excessive CGRP activity that characterizes obese individuals, even though increased neuropeptide release in patients with trigeminal overactivation appears to be associated with a favorable response to trigeminal targeted treatments [25-27]. Therefore, weight loss techniques may be helpful in improving these patients' anti-CGRP mAbs responsiveness [15].

Fifty-five (54%) of the patients had a reduction of at least 50% in MMDs after three months of treatment. In the study by Dodick et al. comparisons between ≥50% of responders and non-responders showed that age was substantially greater in responders than in non-responders, while MHD and total past treatment failures were significantly lower [19].

Age, gender, the prevalence of drug misuse, and the number of prior preventive therapies tried did not correlate with response in an erenumab study. However, the non-responders had a considerably greater mean baseline number of headache days [18].

Among the associated features and characteristics of migraine, the presence of unilateral headache and osmophobia were significantly associated with a higher reduction in MHD. The presence of unilateral headache (OR=3.94(95%CI=1.26-12.33)) was found to be significantly associated with ≥50% reduction in MHD at two months and at four months. According to Nowaczewska et al., mAb responsiveness ≥50% was inversely associated with vascular malformation in the right middle cerebral artery (MCA), negative family history, while positively associated with localised unilateral pain and Headache Impact Test (HIT-6) score [17]. According to Barbanti et al.'s study, a combination of trigeminal sensitization symptoms (unilateral pain (UP) + unilateral cranial autonomic symptoms (UAs)) is the most accurate predictor of ≥50% and ≥75% responses to anti-CGRP mAbs in high-frequency episodic migraine (HFEM) [15].

While there was no difference in the prevalence of phonophobia, nausea, or vomiting, the presence of photophobia was significantly higher among the nonresponders. In the study by Dodick et al. there was no significant difference in the presence of unilateral pain and aura between responders and nonresponders [19].

Erenumab responsiveness was inversely associated with psychiatric comorbidities and had positive association with migraine frequency at baseline, dopaminergic symptoms, and unilateral pain localization in HFEM [21]. In HFEM, cutaneous allodynia was positively correlated with responsiveness to erenumab (OR: 5.44, 95% CI: 1.52-19.41; p=0.009). ≥50% responsiveness was negatively associated with psychiatric comorbidities and previous treatment failures and positively associated with male gender and baseline migraine frequency in CM patients [14].

Raffaeli et al. determined the clinical features of CGRP(-R) mAb super-responders (SR) and absolute non-responders (NR) in their retrospective investigation. Compared to NR, SR patients had a statistically higher likelihood of experiencing migraine attacks with the characteristic symptoms of unilateral localization, pulsing nature, nausea, and vomiting; only vomiting reached statistical significance. In contrast, NR had depression, medication-overuse headaches (MOH), and CM twice as frequently as SR [28].

There was no significant difference (p=0.243) in the reduction of MHD between the two different types of intervention (fremanezumab 225/month and galcanezumab 240-120/month) given for treatment of migraine. However, the type of treatment was as per the choice of the patients. The three anti-CGRP mAbs were found to be equally effective in two recent meta-analyses, which included seven and 18 randomized controlled trials (RCTs), respectively [29,30].

Limitations

The study's uniqueness is in its attempt to identify the determinants of response to the two mAbs that are accessible in Brazil for the treatment of episodic migraine: galcanezumab and fremanezumab. In the research center, very few individuals receive monotherapy as preventative treatment for migraines. The single-center design and patient selection criteria may introduce selection bias, potentially limiting generalizability. However, an advantage is that in addition to mAbs, the majority of migraineurs from tertiary centers in Brazil also take at least one conventional pharmacological treatment, which was not the case with our series. The history of employing various treatment approaches and the distinctive profile of seeing multiple doctors prior to our care are the reasons for this reality in most patients attending tertiary centers for headache sufferers. Only patients who had not utilized any additional preventive measures in the preceding six months were included, nevertheless. Another limitation of the study was the lack of evaluation of the effect of comorbidities, short follow-up period, and absence of a control group and their treatments on response to treatment with mAbs.

## Conclusions

Only normal BMI was found to be significantly associated with a reduction in migraine frequency ≥50%, whereas normal BMI, the presence of unilateral headache, and osmophobia were significantly associated with the mean reduction in the MHD. Longer follow-up periods with additional factors evaluated to predict treatment response, such as genetic markers and medication adherence, are advised by future large-scale studies.
